# Sino-Austrian High-Tech Acupuncture Network: Annual Report 2017

**DOI:** 10.3390/medicines5010005

**Published:** 2018-01-12

**Authors:** Gerhard Litscher

**Affiliations:** Head of the Research Unit for Complementary and Integrative Laser Medicine, of the Research Unit of Biomedical Engineering in Anesthesia and Intensive Care Medicine, and of the TCM Research Center Graz, Medical University of Graz, Auenbruggerplatz 29, 8036 Graz, Austria; gerhard.litscher@medunigraz.at; Tel.: +43-316-385-13907

**Keywords:** high-tech acupuncture, network, annual report, China, Austria

## Abstract

The Sino-Austrian High-Tech Acupuncture Research Network was founded in 2005 and has been growing ever since. The network comprises many partners from China and is highly involved in research and education activities. This report introduces the network’s activities in the year 2017.

The High-Tech Acupuncture Network was founded in 2005 by Professor DDr. Gerhard Litscher from the Medical University of Graz (Traditional Chinese Medicine (TCM) Research Center Graz) and comprises many partners from China (see Figure 1).

In 2017, the Sino-Austrian High-Tech Acupuncture Network has grown very fast. In the following, milestones from the year 2017 are listed chronologically and at the end of the report recent literature from the network is cited [1,2,3,4,5,6,7,8,9,10,11,12,13,14,15] (see also Figure 2, Figure 3, Figure 4, Figure 5, Figure 6, Figure 7, Figure 8, Figure 9, Figure 10, Figure 11, Figure 12, Figure 13, Figure 14, Figure 15, Figure 16, Figure 17, Figure 18, Figure 19, Figure 20, Figure 21, Figure 22, Figure 23, Figure 24, Figure 25, Figure 26, Figure 27, Figure 28, Figure 29, Figure 30, Figure 31, Figure 32, Figure 33 and Figure 34):

**8 January 2017:** World Health Organization (WHO): Professor Gerhard Litscher has been invited by the WHO (Traditional and Complementary Medicine) in Geneva to be an official participant in the following two working groups:

WHO Benchmark for Practice in Acupuncture
WHO Benchmark for Practice in Tuina

**16 February 2017:** Acupuncture Student Lecture, Professor Liang Fengxia, Graz, Austria.

Professor Liang Fengxia, Associate Dean, Acupuncture and Moxibustion Orthopaedic College and Deputy Director, Acupuncture and Moxibustion Institute at the Hubei University of Chinese Medicine, performed a 3-hour student lecture at the Medical University of Graz (Special TCM Module (lecture, G. Litscher), coordinator Dr. Andrea Pribyl).

**12 March 2017:** Eurasia Pacific Uninet (EPU). Joint project with the Hubei University of Chinese Medicine, Wuhan, China and the Medical University of Graz, Graz, Austria.

Established in 1958, the Hubei University of Chinese Medicine has three secondary schools, four affiliated hospitals, four State-Level (highest level) Medical Research Centers, and 10 Research Institutions. The university occupies 0.65 km^2^ with a total construction of about 470,000 m^2^. The university has 15 departments, 17 specialties for the bachelor’s degree and 9 specialties for professional training, 19 specialties for the master’s degree and 12 specialties for the doctor degree, and more than 60 bases for clinical practice, including 6 affiliated hospitals, and 21 State-Level, Province-Level, or College-Level laboratories for teaching. At present, the university has about 15,000 students.

Acupuncture is one of the key disciplines of research of this renowned university. The Medical University of Graz (Professor Gerhard Litscher) works in close cooperation with the Hubei University of Chinese Medicine (with Professor Wang Hua and Professor Liang Fengxia) on the topic of high-tech acupuncture research. Within the few last years, several joint SCI/PubMed-listed publications have been published. Professor Litscher from the Medical University of Graz is Visiting Professor at the Hubei University of Chinese Medicine and at the Hubei Provincial Collaborative Innovation Center of Preventive Treatment by Acupuncture and Moxibustion (Director: Professor Wang Hua). The cooperation is also supported by the Austrian Ministry of Science, Research, and Economy and by Eurasia Pacific Uninet.

**21 May 2017:** Editor’s Meet in Beijing: Medicines, Beijing, China (G. Litscher: Editor-in-chief of Medicines).

**21 May 2017:** World Federation of Chinese Medicine Societies, Beijing, China. Professor G. Litscher: Executive Council Member (December 2016–December 2020). Topic: Heart Rate Variability.

**22 May 2017:** Sino-Austrian Project Meeting 2017. Sino-Austrian TCM Research on Lifestyle Related Diseases, Beijing, China.

**23 May 2017:** Tianjin University of Traditional Chinese Medicine, Acupuncture Seminar, Tianjin, China.

**24 May 2017:** Beijing Tongren Hospital affiliated to Capital Medical University (CMU), Beijing, China, Project Discussion: Department of Anesthesiology.

**24 May 2017:** China Academy of Chinese Medical Sciences (CACMS), Beijing, China.

**25 May 2017:** Peking University Health Science Center and Eurasia Pacific Uninet (EPU) PhD Interviews: Professors. D. Rausch and G. Litscher, Beijing, China.

**26 May 2017:** Capital Medical University (CMU): Beijing Hospital of TCM, Beijing, China.

**9–10 June 1017:** 12th International ISLA (International Society for Medical Laser Applications) Congress, Beverungen, Germany.

**9 June 2017:** Meeting with Representatives from WALT (World Association of Laser Therapy), Lauenförde, Germany.

**24–25 June 2017:** Interdisciplinary Acupuncture Symposium V, Athens, Greece: Science focuses on Pain.

**26 June 2017:** China Academy of Chinese Medical Sciences at the Medical University of Graz, Graz, Austria.

Established in 1955, China Academy of Chinese Medical Sciences (CACMS) is China’s largest comprehensive research institute combining scientific research, medical treatment, and teaching that is directly under the State Administration of TCM (Traditional Chinese Medicine). It boasts various disciplines, advanced equipment, and great research strength and has under it 17 research institutes, six medical institutions, one graduate school, two branch schools, two pharmaceutical companies, and publishing houses of ancient books on Chinese medical science. Besides, it is a founder of 18 kinds of academic journals on Chinese medical science.

What is especially worth mentioning is the achievement in artemisinin research, which has provided a powerful weapon for humans against malaria, saved hundreds of thousands of lives, and made tremendous contributions to human health. Thus, CACMS was awarded a Lasker Medical Research Award and in 2015 the Nobel Prize in Medicine.

Acupuncture is one of the key disciplines of research of this renowned university. The Medical University of Graz works in close cooperation with China Academy of Chinese Medical Sciences on the topic of high-tech acupuncture research. Within the last 12 years, more than 65 joint SCI/PubMed-listed publications have been published with CACMS together. Professor Litscher from the Medical University of Graz is Visiting Professor at the Institute of Acupuncture and Moxibustion at CACMS. This appointment was renewed last year during the last visit of CACMS to the Medical University of Graz with President Professor Zhang Boli.

**2 July 2017:** 10th Anniversary of the Traditional Chinese Medicine (TCM) Research Center Graz, Austria, Europe.

From acupuncture to the many hundreds of different medical herbs: Traditional Chinese Medicine (TCM) is booming. In addition, it is effective: TCM has been practised successfully for more than 4000 years, and the Western demand for something to complement classical Western medicine has been increasing for years. Graz is going to play a central role in TCM research: the “TCM Research Center Graz” was founded in early March 2007 by Karl-Franzens-University Graz and the Medical University Graz, and subsequently became a competence center that is unique worldwide.

Dealing with acupuncture and Chinese medical herbs has a long tradition in Graz: Professor Rudolf Bauer, Head of the Institute of Pharmaceutical Sciences at Karl-Franzens-University, has been researching the active pharmaceutical ingredients and quality of Chinese medicinal herbs now for 26 years. Professor Gerhard Litscher, Head of the Research Unit of Biomedical Engineering in Anesthesia and Intensive Care Medicine at the Medical University of Graz, has dedicated himself to the research of acupuncture using the latest high-tech methods for 20 years. For both, scientific work is the basis for the modernization of TCM: “TCM is a kind of medicine that can be evaluated scientifically”, says Rudolf Bauer, “it possesses comprehensible diagnostic methods and uses precise and controllable therapies”. So, all research work is done based on scientific methods. Gerhard Litscher: “We are interested in basic research and those aspects of TCM that have not been given much attention so far; for example, the quantification of new acupuncture techniques, such as the painless laser needle acupuncture and electro acupuncture. Possible effects of acupuncture in combination with other methods are also subject to scientific research”.

A scientific report summarizes some of the research activities within the last 10 years and the chairmen of the center would like to use the opportunity to thank everyone for their generous support of the TCM Research Center Graz.

**8 August 2017:** Lecture about High-Tech Acupuncture at Eu Yan Sang Intern. Ltd.—Singapore, Republic of Singapore.

**10–12 August 2017:** 9th International Symposium on Auriculotherapy, Singapore, Republic of Singapore. Report published in Medicines [12].

**11–15 September 2017:** Acupuncture Congress: Germany, Timmendorfer Strand, Germany.

Gerhard Litscher (Lecture: Innovations in the Field of Acupuncture Research; Workshop: Scientific Publishing).

**23 September 2017:** Project Meeting: China Academy of Chinese Medical Sciences, Beijing, China.

**25–27 September 2017:** 4th Annual World Congress (BIT) of High-Tech Acupuncture and Integrative Medicine (HTA&IM): 2017, and 2nd Annual World Congress (BIT) of Modern Chinese Medicine: 2017, Xi’an, China.

Professor Gerhard Litscher, Chairman of the two World Congresses, Sheraton Hotel, Xi’an, China, 400 participants at 4 congresses (opening ceremony), 110 participants at HTA&IM (57 speakers from 12 countries). Report published in Medicines [11].

**27 September 2017:** Capital Medical University, Beijing Hospital of TCM, China.

**27 September 2017:** People’s Medical Publishing House, Beijing, China.

**28 September 2017:** Editor’s Meet in Beijing: Medicines (Pubmed-listed since September 2017), Beijing, China.

**28 September 2017:** Beijing Tongren Hospital affiliated to Capital Medical University: Project Meeting Swissotel, Beijing, China, Project Discussion: Department of Anesthesiology and Department of Acupuncture and Moxibustion.

**17 October 2017:** Project Cooperation: People’s Liberation Army General Hospital, Beijing University of Chinese Medicine, and Medical University of Graz, Graz, Austria, Project Discussion.

**9–11 November 2017:** 1st World Congress (BIT) of Biomedical Engineering: 2017, Xi’an, China. Hilton, Xi’an, China, 380 participants, Report published in *Medicines* [10].

**30 November 2017:** Beijing Hospital of TCM affiliated to Capital Medical University (CMU), Beijing, China. Project Meeting (EPU-Project 05/2017).

**1 December 2017:** Editorial Board Meeting in Beijing: Medicines (Pubmed-listed since September 2017), Beijing, China.

**2–4 December 2017:** The 9th General Assembly of the World Federation of Acupuncture: Moxibustion Societies (WFAS) and WFAS Congress, Beijing, China.

**4 December 2017:** The 5th Editorial Board of the World Journal of Acupuncture: Moxibustion. Beijing, China.

**5 December 2017:** Cooperation between the Medical University of Graz (TCM Research Center) and Hubei University of Chinese Medicine, Wuhan, China.

**6 December 2017:** Huazhong University of Science and Technology, Tongji Medical College, School of Nursing, Wuhan, China.

## Figures and Tables

**Figure 1:**
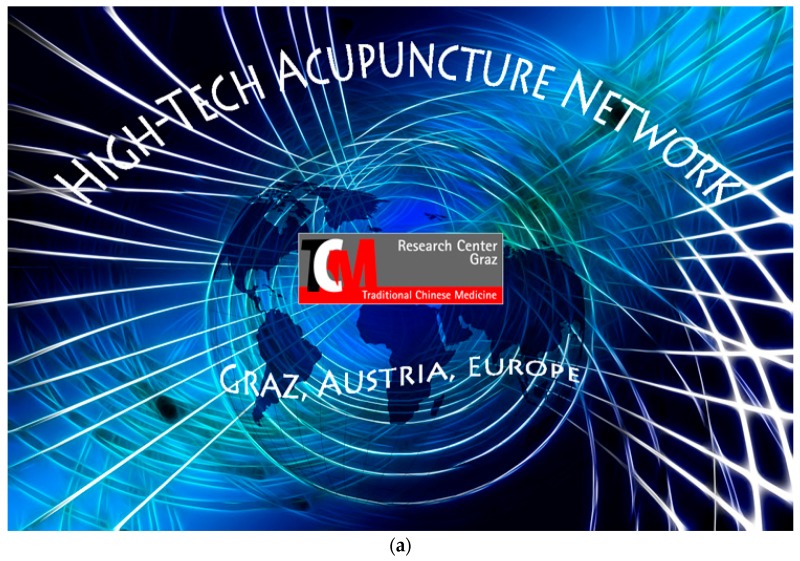

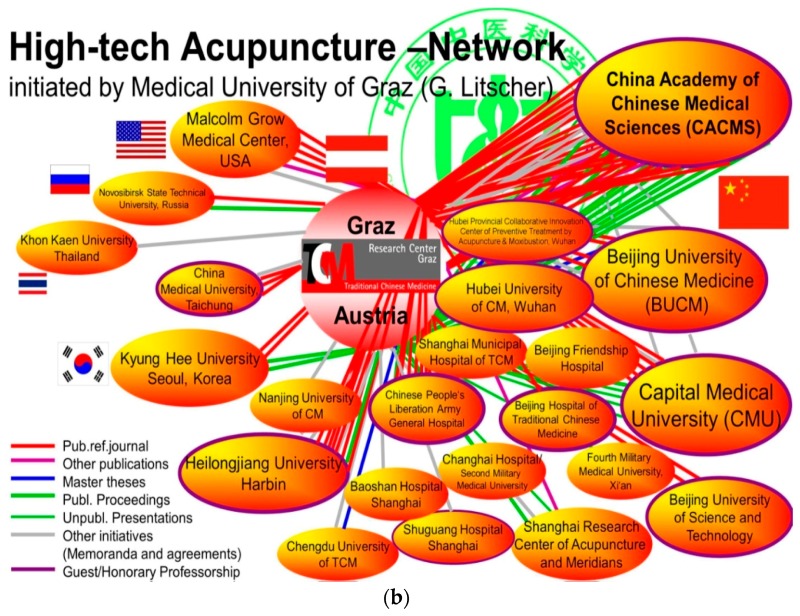
High-tech acupuncture network initiated in Graz (**a**) and current partners, including scientific information (**b**). CM: Chinese Medicine; TCM: Traditional Chinese Medicine.

**Figure 2:**
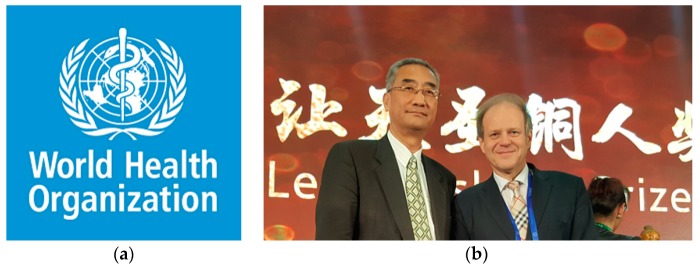
World Health Organization (WHO), Geneva, Switzerland, 2017 (**a**) Dr. Zhang Qi, WHO Coordinator for Traditional, Complementary, and Integrative Medicine (left) with Professor Gerhard Litscher (**b**).

**Figure 3:**
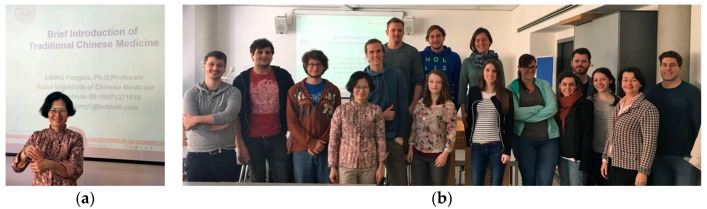
Professor Liang Fengxia (**a**) in Graz, Austria, 16 February 2017. Student lecture from Professor Liang at the Medical University of Graz. Coordinator: Dr. Andrea Pribyl (2nd on the right side) (**b**).

**Figure 4:**
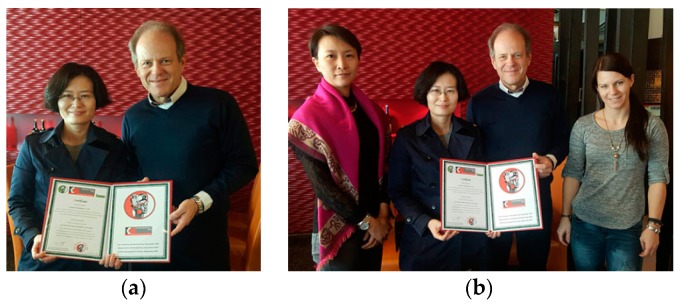
Professor Liang Fengxia and Professor Gerhard Litscher (**a**) From left to right: PD Dr. Lu Wang, Professor Liang Fengxia, Professor Gerhard Litscher, and Dr. Daniela Litscher (**b**) Graz, Austria, 2017.

**Figure 5:**
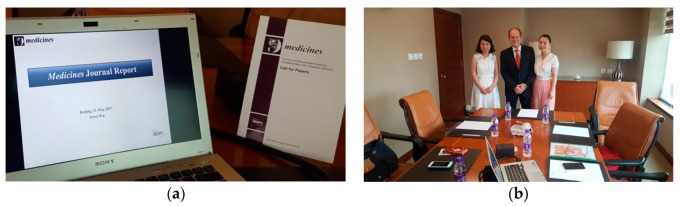
Medicines Editor’s Meeting in Beijing, China, 21 May 2017. Presentation of a report by the Managing Editor Mrs. Jewel Wu (**a**). From left to right: Mrs. Chao Xiao, Section Leader, Professor Gerhard Litscher, Editor-in-chief, and Mrs. Jewel Wu, Managing Editor (**b**).

**Figure 6:**
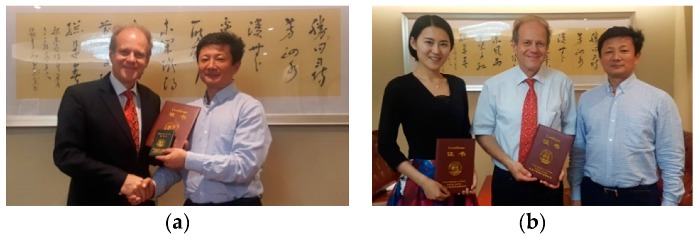
Chairman Professor Wu Yongjian (Peking Union Medical College) with Professor Gerhard Litscher (**a**) and Wang Huan MM (General Hospital of Chinese People’s Liberation Army), Professor Gerhard Litscher, and Professor Wu Yongjian (**b**) in Beijing.

**Figure 7:**
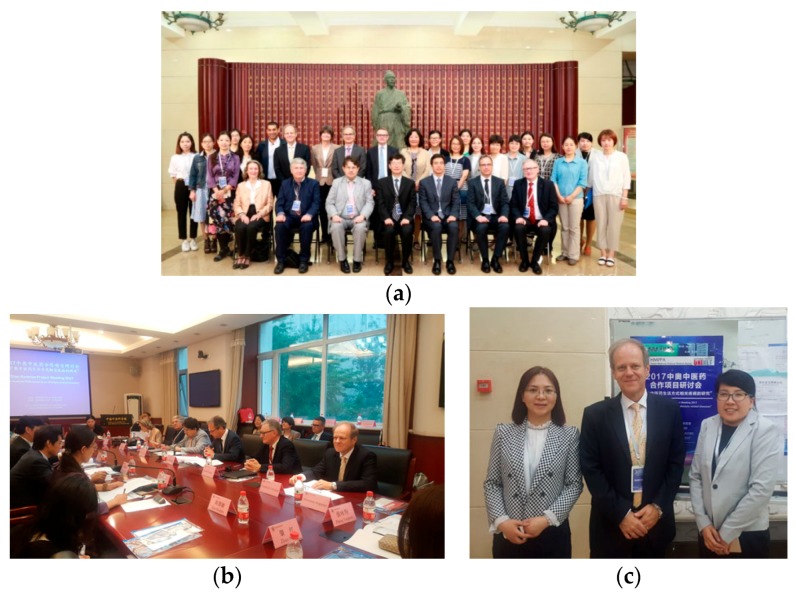
Sino-Austrian project meeting at China Academy of Chinese Medical Sciences, Beijing, China, 22 May 2017 (**a**–**c**). From left to right: Professor Gao Xinyan, Professor Gerhard Litscher, and Dr. Liu Kun (**c**).

**Figure 8:**
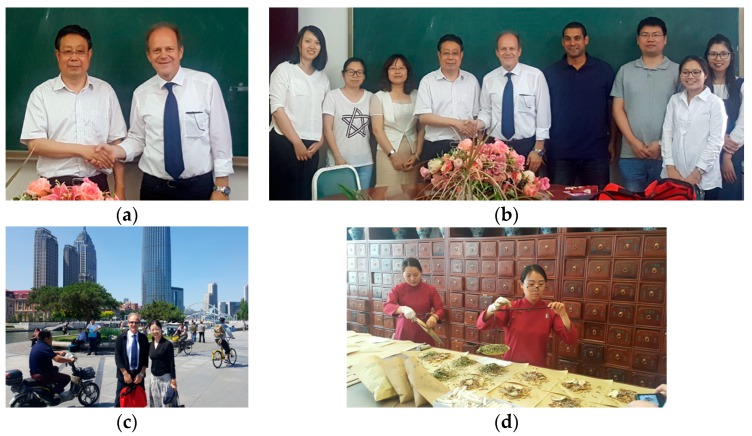
Tianjin University of Chinese Medicine (TUCM). Professor Guo Yi with Professor Gerhard Litscher (**a**). Acupuncture Seminar (**b**). Tianjin, China. Professor Song Ping, China Academy of Chinese Medical Sciences with Professor Gerhard Litscher (**c**), TUCM (**d**), 23 May 2017.

**Figure 9:**
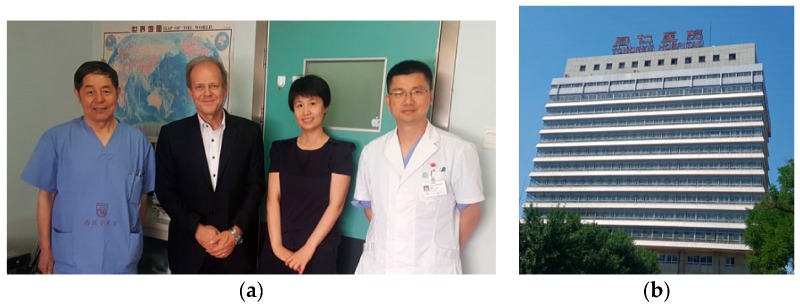
(**a**) Professor Zhang Bingxi (left), Professor Pan Chuxiong (Head of the Department of Anesthesiology), Dr. Sun Yanxia (middle), and Professor Gerhard Litscher. (**b**) Tongren Hospital affiliated to Capital Medical University (CMU), Beijing, China, 24 May 2017.

**Figure 10:**
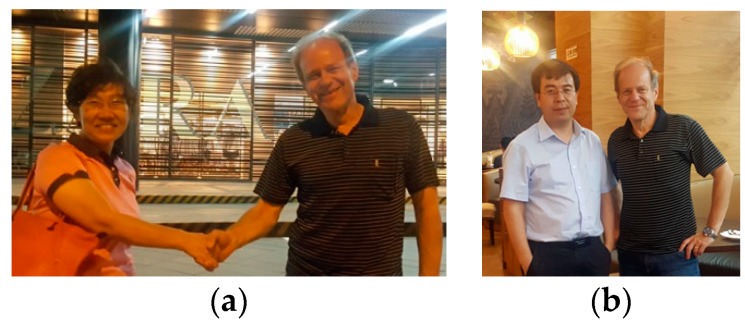
Professor Gerhard Litscher with Professor Jing Xianghong, Director of the Institute of Acupuncture and Moxibustion, China Academy of Chinese Medical Sciences (CACMS) (**a**) and Dr. Wang Guangjun, CACMS; (**b**) Beijing, China, 24 May 2017.

**Figure 11:**
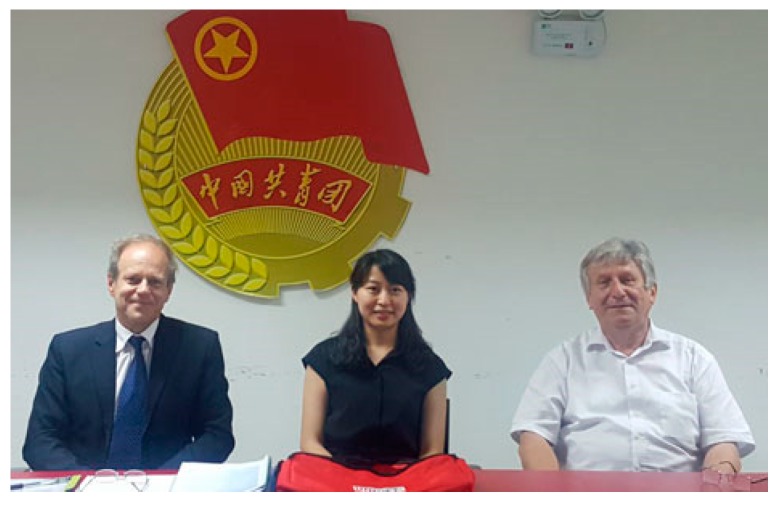
Peking University, Beijing, China, 25 May 2017. From left to right: Professor Gerhard Litscher, Eurasia Pacific Uninet (EPU) scholarship applicant, and Professor Wolf-Dieter Rausch, President of EPU.

**Figure 12:**
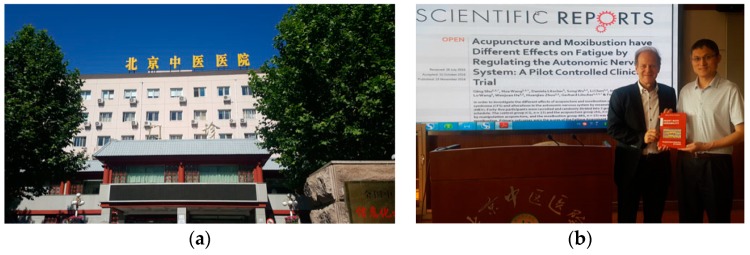
Project Discussion at the Beijing Hospital of TCM affiliated to CMU (**a**) Professor Liu Cun Zhi (right) and Professor Gerhard Litscher (**b**) Beijing, 26 May 2017.

**Figure 13:**
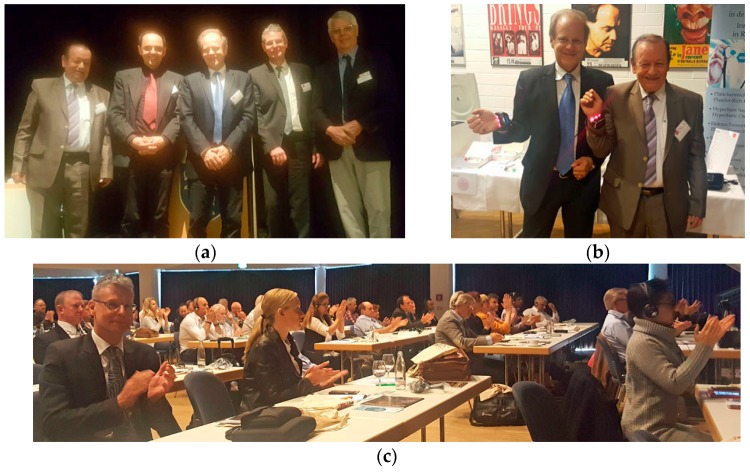

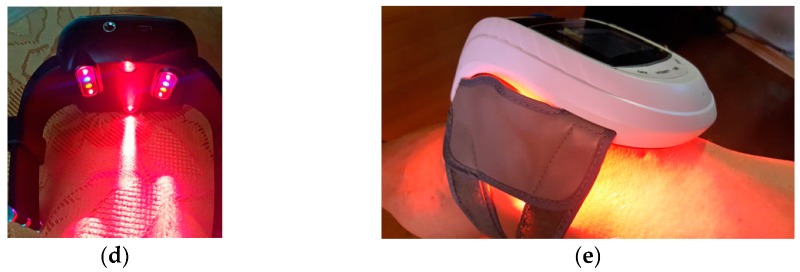
International ISLA (International Society for Medical Laser Applications) Congress, Beverungen, Germany, June 9, 2017 (**a**–**e**). (**a**) From left to right: Dr. Michael H. Weber (ISLA President), Dr. Michael Weber, Professor Gerhard Litscher (ISLA President), Dr. Volkmar Kreisel (ISLA Vice-President), Dr. Michael Grandjean (ISLA Vice President). Presidents of ISLA (International Society for Medical Laser Applications) and of the Congress: Professor G. Litscher (left) and Dr. M. H. Weber (**b**). Laser stimulation equipment for the wrist (**d**) and the knee (**e**).

**Figure 14:**
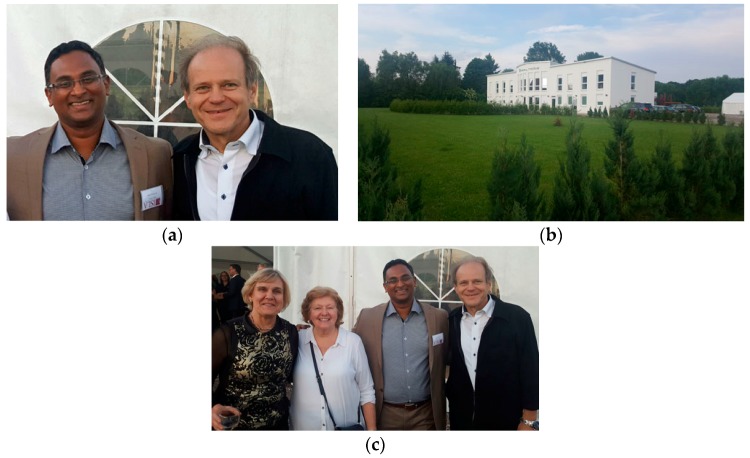
President elect of WALT, Professor P.R. Arany, University at Buffalo, New York and President of ISLA (International Society of Medical Laser Applications), Professor Gerhard Litscher (right) (**a**,**c**). Representatives from WALT, Dr. Ann Liebert (Sydney, Australia; left), Professor Juanita J. Anders (Bethesda, Maryland, USA), Professor P.R. Arany, and Professor G. Litscher (**c**) in Lauenförde (**b**), Germany, 9 June 2017.

**Figure 15:**
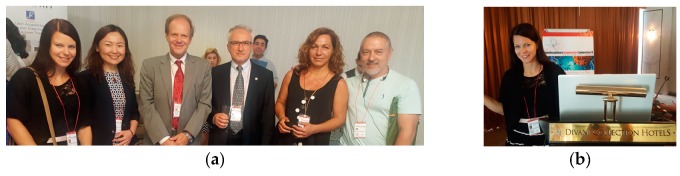

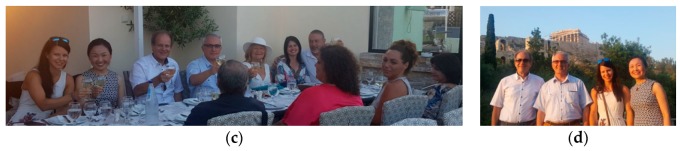
Interdisciplinary Acupuncture Symposium V, Athens, Greece (**a**–**d**). (**a**) From left to right: Dr. Daniela Litscher (Austria), Professor Rong Peijing (China), Professor Gerhard Litscher (Austria), Professor Ilhan Öztekin (Turkey), Dr. Konstantina Theodoratou (Greece), Dr. Antonio C. Sant’Ana (Brazil).

**Figure 16:**
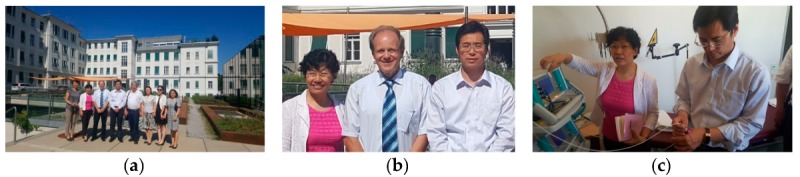
(**a**–**c**) Delegation from China Academy of Chinese Medical Sciences at the Medical University of Graz. (**b**) From left to right: Professor Jing Xianghong, Deputy Director of the Institute of Acupuncture and Moxibustion, China Academy of Chinese Medical Sciences, Professor Gerhard Litscher, and Professor Yang Longhui, Vice President of China Academy of Chinese Medical Sciences.

**Figure 17:**
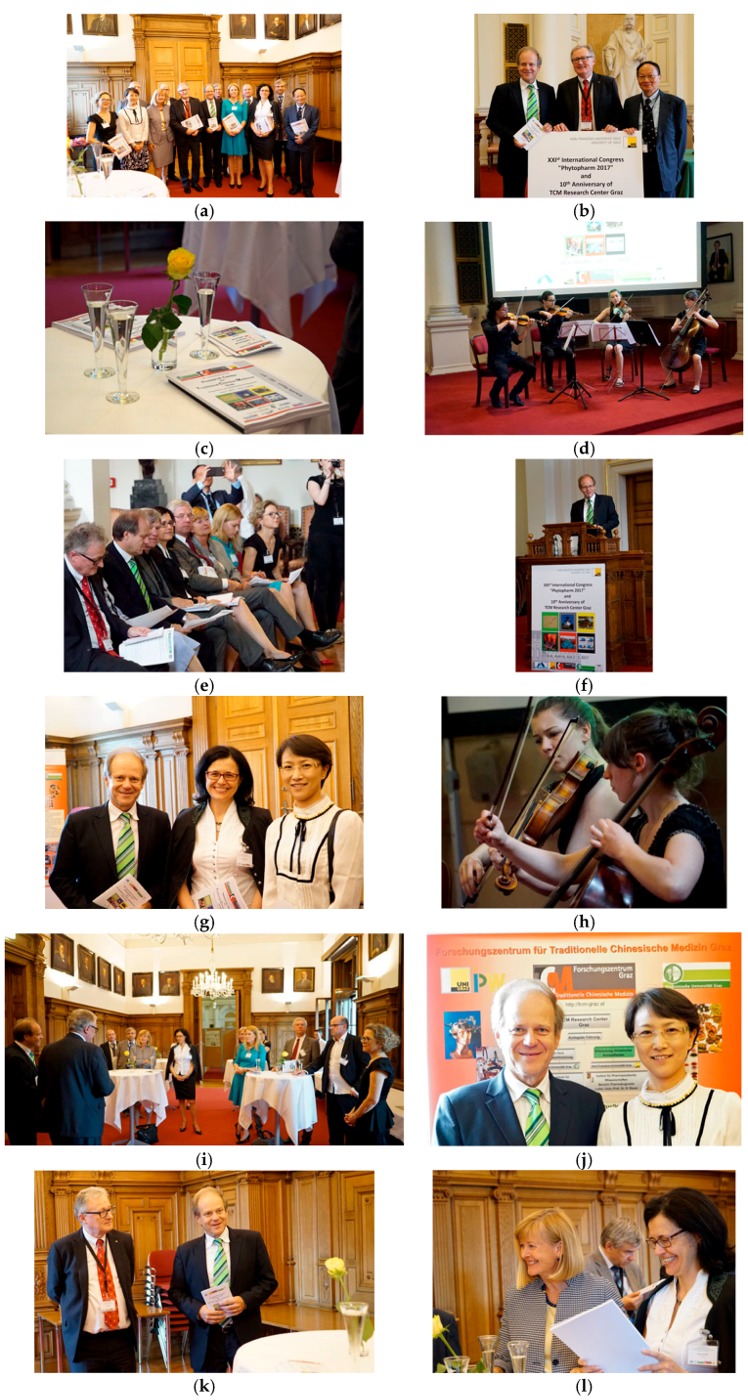

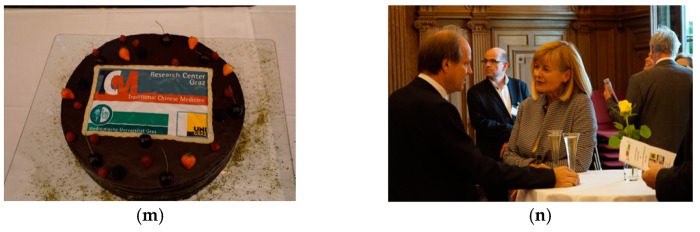
Tenth Anniversary of the TCM Research Center Graz (**a**–**n**). The interuniversity TCM Research Center Graz was founded in 2007 under the leadership of Professor Rudolf Bauer (**b**, **middle**), Head of the Institute of Pharmaceutical Sciences, Department of Pharmacognosy, Karl-Franzens-University Graz and under the leadership of Professor Gerhard Litscher (**b**, **left**), Head of the Research Unit of Biomedical Engineering in Anesthesia and Intensive Care Medicine, Medical University Graz.

**Figure 18:**
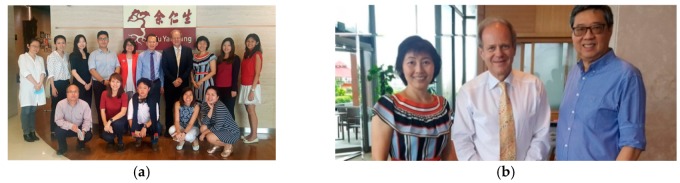

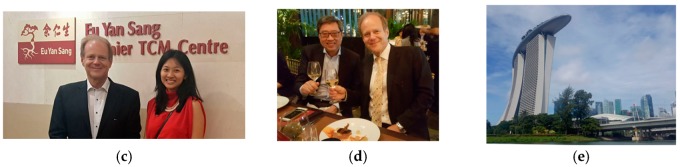
Eu Yan Sang (**a**–**d**) Australia–China–Hong Kong–Indonesia–Macau–Malaysia–Singapore–USA. (**b**) From left to right: Dr. Caryn Peh (Managing Director), Professor Gerhard Litscher, and Dr. Richard Y. M. Eu (Chief Executive Officer). (**c**) Professor Gerhard Litscher with Dr. Alicia Lim. Singapore (**e**) 8 August 2017.

**Figure 19:**
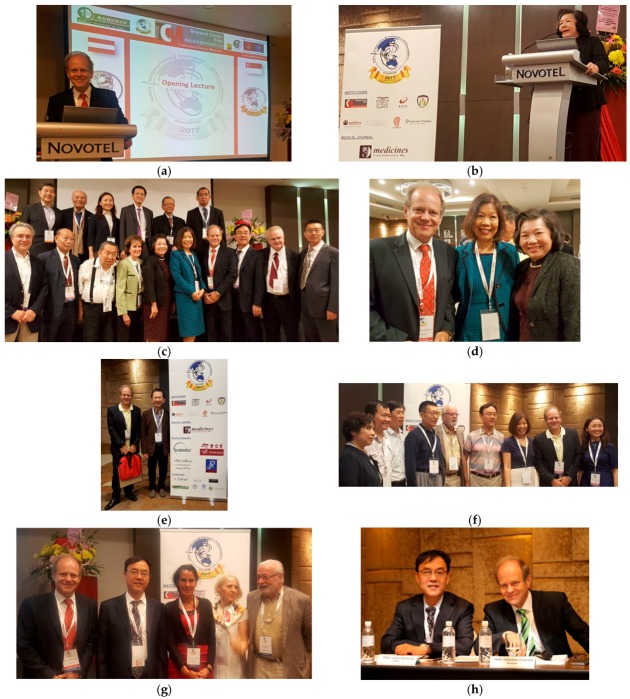

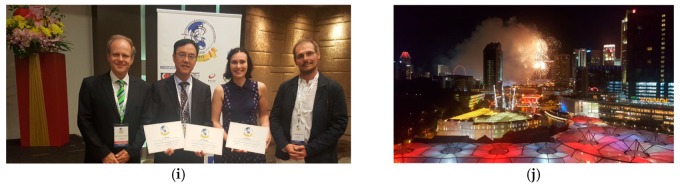
(**a**–**j**) Ninth International Symposium on Auriculotherapy, Singapore, Republic of Singapore. (**a**) Opening Lecture by Professor Gerhard Litscher. (**b**) Mrs. Dr. Yu-Foo Yee Shoon, former Minister of State and Chairwoman of the TCM Board Singapore. (**c**) Organizing Committee. (**d**) Dr. Im Quah-Smith (Congress Chair, Australia, middle), Gerhard Litscher (Keynote Speaker and Member of the Organizing Committee), and Dr. Yu-Foo Yee Shoon. Singapore, 10–12 August 2017.

**Figure 20:**
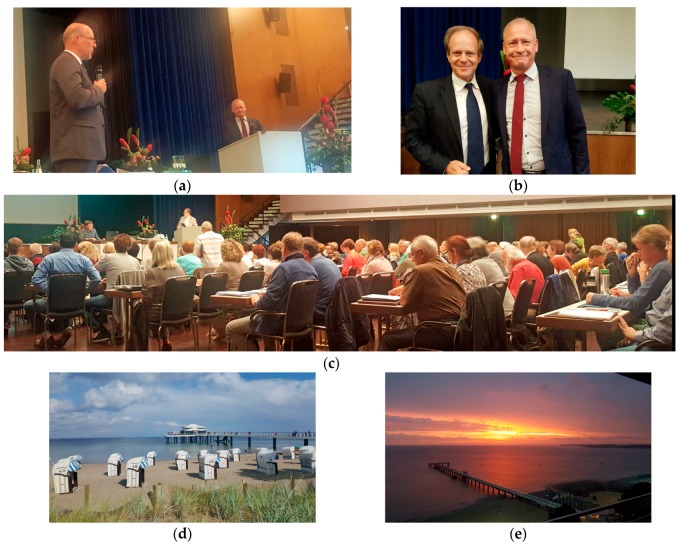
Acupuncture Congress: Germany (**a**–**e**). (**a**) Dr. Bernd Ramme (Chair of the DAA e.V., left) and PD Dr. Dominik Irnich (Chair of DÄGfA e.V.); (**b**) Professor Gerhard Litscher and PD Dr. Dominik Irnich. Timmendorfer Strand (**d**,**e**). Germany, 12 September 2017.

**Figure 21:**
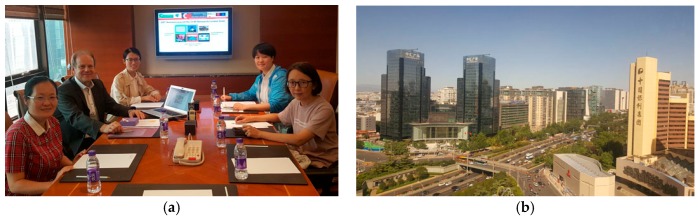
(**a**) From left to right: Associate Professor He Wei, Professor Gerhard Litscher, Assistant Professor Zhang Xiaoning, Assistant Professor Wang, and Associate Professor Wang Xiaoyu. Beijing (**b**) China, 23 September 2017.

**Figure 22:**
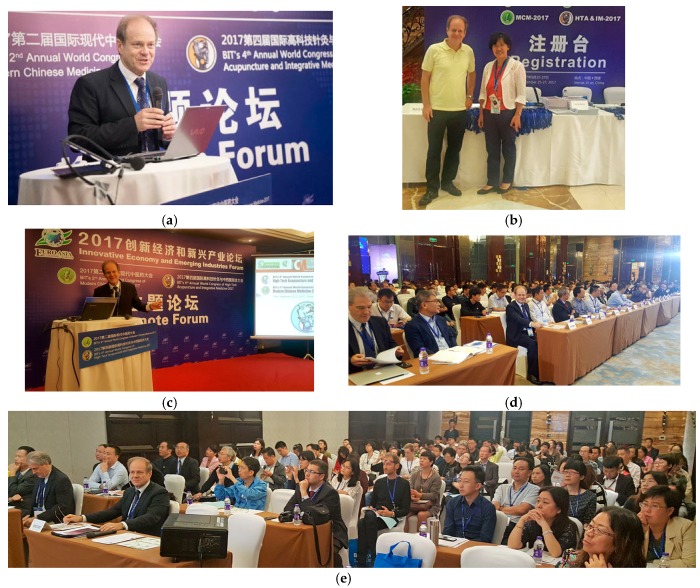

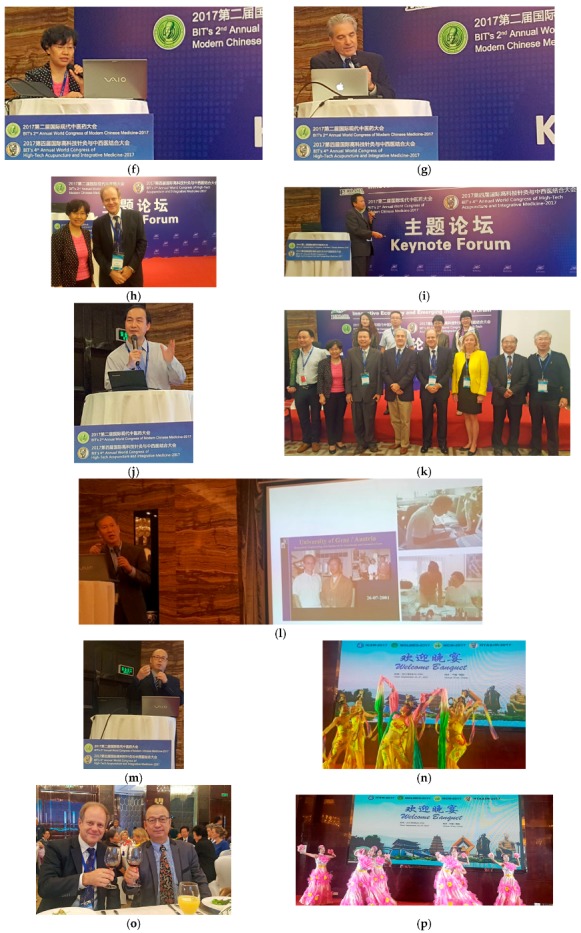

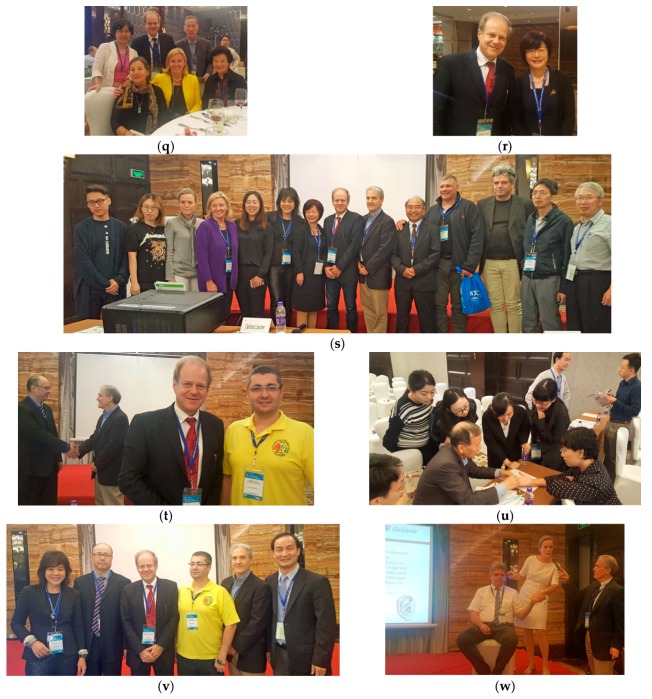
Fourth Annual World Congress (BIT) of High-Tech Acupuncture and Integrative Medicine: 2017 and 2nd Annual World Congress (BIT) of Modern Chinese Medicine: 2017, Xi’an, China (**a**–**w**). 25–27 September 2017.

**Figure 23:**
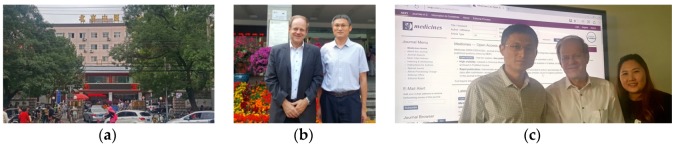
(**a**) Beijing Hospital of TCM affiliated to CMU; (**b**) Professor Gerhard Litscher (left) and Professor Liu Cun Zhi; (**c**) From left to right: Professor Liu Cun Zhi, Professor Gerhard Litscher, and Mirim Kim. Beijing, China, 27 September 2017.

**Figure 24:**
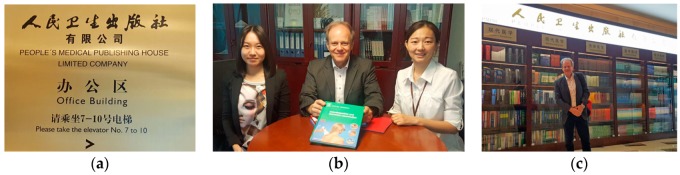
People’s Medical Publishing House (**a**–**c**). (**b**) From left to right: Cheryl Zhang (International Dept., People’s Medical Publishing House, Beijing, China), Professor Gerhard Litscher, and Emma Zhang (Senior Product Development Executive, Elsevier, Beijing, China). Beijing, China, 27 September 2017.

**Figure 25:**
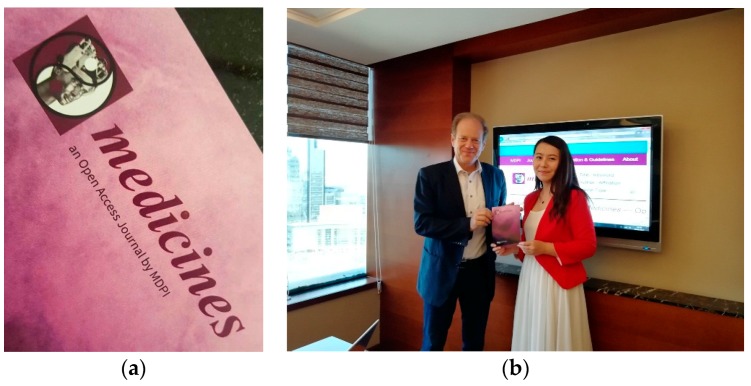
(**a**) Medicines journal flyer; (**b**) Professor Gerhard Litscher: Editor-in-chief of Medicines and Ms. Jewel Wu, former Managing Editor, MDPI Haidian Office, Beijing, China, 28 September 2017.

**Figure 26:**
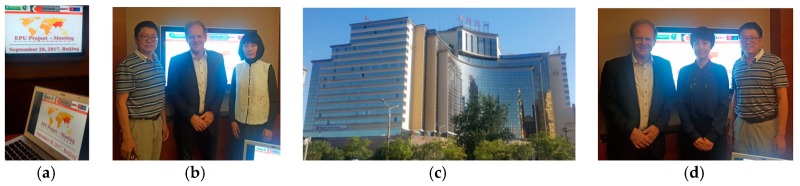
Project Meeting (**a**) at Swissotel, Beijing (**c**). (**b**) From left to right: Professor James Chen, Director of TCM and Acupuncture Department of Beijing Tongren Hospital, Professor Gerhard Litscher, and Dr. Xiaojuan Shang, Acupuncture Department of Tongren Hospital. (**d**) From left to right: Professor Gerhard Litscher, Dr. Sun Yanxia, Department of Anesthesiology of Beijing Tongren Hospital, and Professor James Chen. Beijing, China, 28 September 2017.

**Figure 27:**
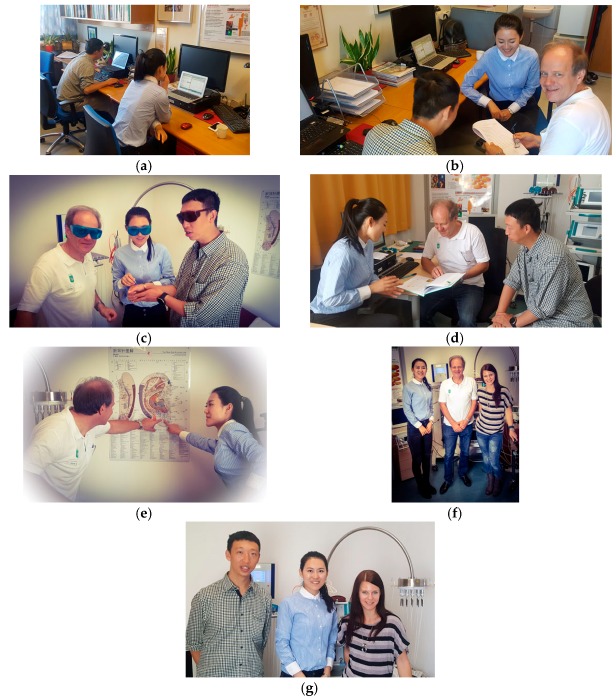
Project Cooperation: People’s Liberation Army General Hospital, Beijing University of Chinese Medicine, and Medical University of Graz (**a**–**g**). (**d**) From left to right: Huan Wang, MM (People’s Liberation Army General Hospital), Professor Gerhard Litscher, and Dr. Li Guangzong (Beijing University of TCM). (**g**) From left to right: Dr. Li Guangzong, Huan Wang, MM, and Dr. Daniela Litscher. 17 October 2017.

**Figure 28:**
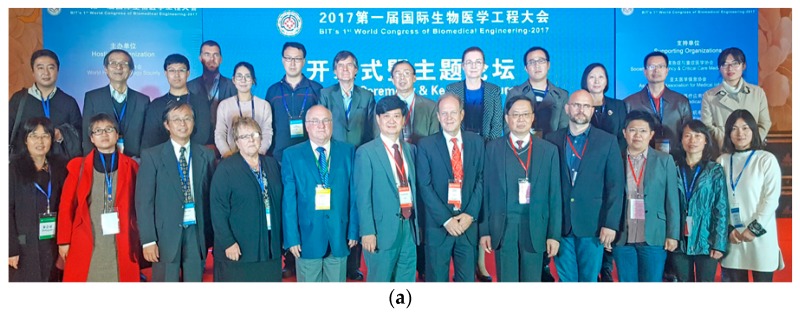

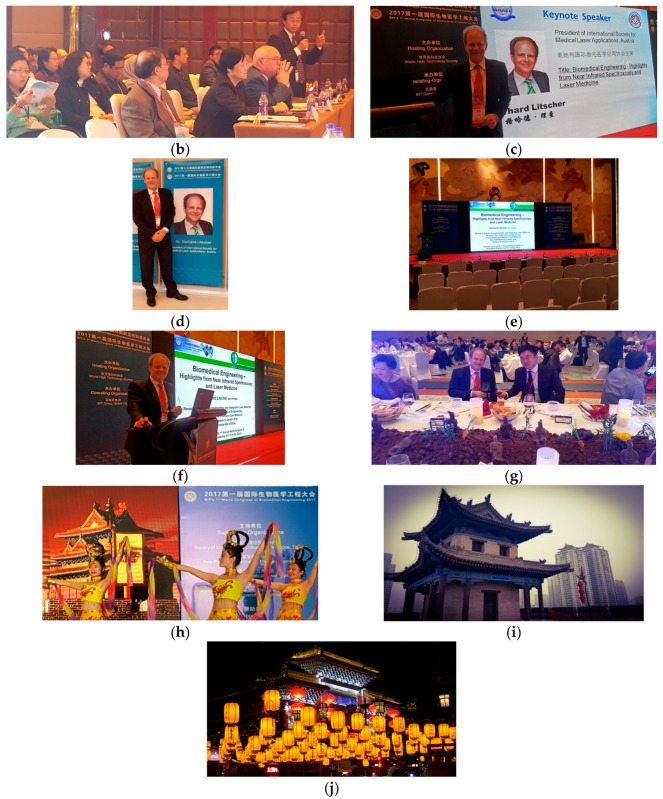
First World Congress (BIT) of Biomedical Engineering (**a**–**j**) 9–12 November 2017. (**a**) Keynote Forum; (**c**,**d**,**f**) Keynote Speaker Professor G. Litscher; (**g**,**h**) Welcome Banquet; (**i**,**j**) Xi’an, China.

**Figure 29:**
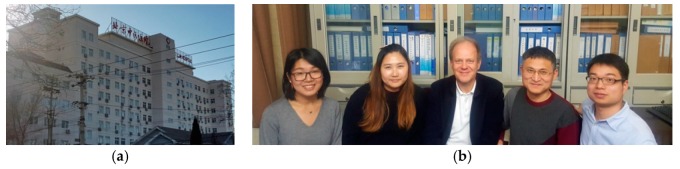
(**a**) Beijing Hospital of TCM affiliated to CMU; (**b**) The research team of the hospital with Professor Gerhard Litscher (middle) and Professor Liu Cun Zhi (middle right) in Beijing, 30 November 2017.

**Figure 30:**
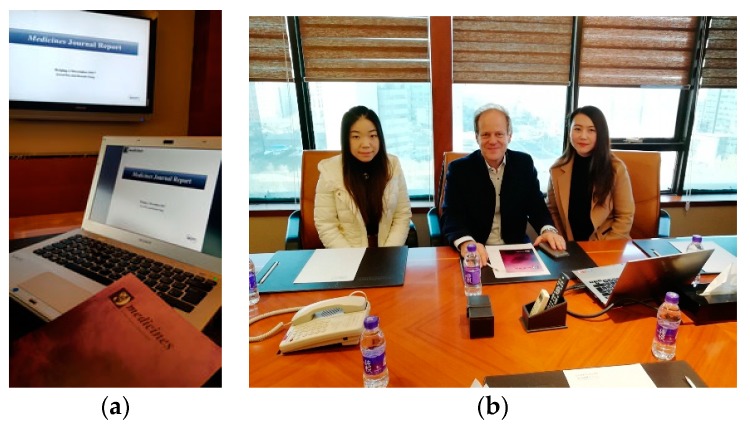
(**a**) Medicines journal report; (**b**) From left to right: Ms. Bonnie Yang, Managing Editor, Professor Gerhard Litscher, Editor-in-chief, and Ms. Jewel Wu, former Managing Editor, Beijing, China, 1 December 2017.

**Figure 31:**
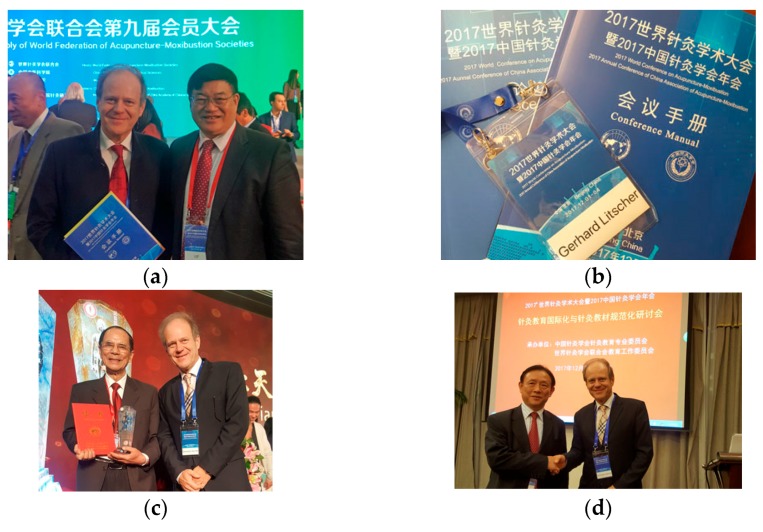

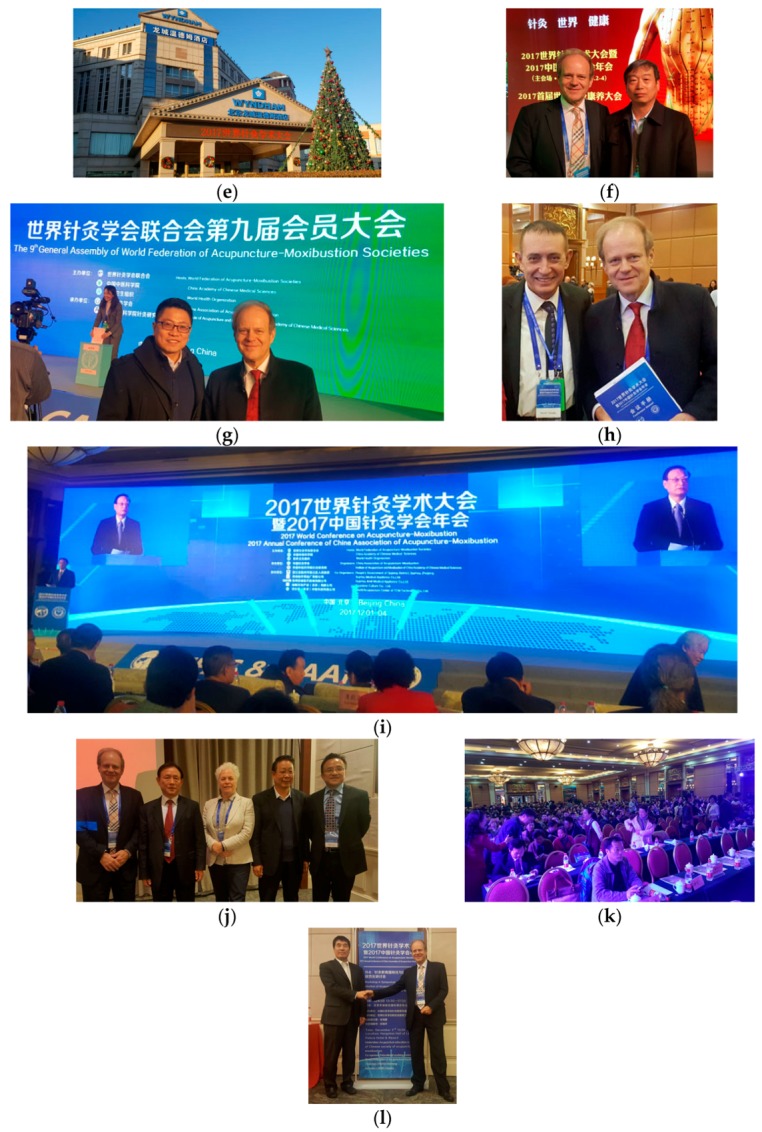
The Ninth General Assembly of the World Federation of Acupuncture: Moxibustion Societies (WFAS) and WFAS Congress (**a**–**l**). (**a**) Professor Liu Baoyan (Chairman of WFAS) and Professor Gerhard Litscher (left); (**c**) Professor Han Ji Sheng (left). (**d**) Professor Wang Hua (left), former President of Hubei University of Chinese Medicine; (**f**) Professor Zhang Weibo (right), China Academy of Chinese Medical Sciences; (**g**) Professor James Chen (left), Director of Traditional Chinese Medicine and Acupuncture Department of Beijing Tongren Hospital; (**h**) Dr. Murat Topoglu (left), President of the Turkish Acupuncture Society; (**i**) Dr. Wang Guoqiang, Honourable Vice Minister; (**j**) From left to right: Professor Gerhard Litscher, Professor Wang Hua, Dr. Judy James, Professor Guo Yi, and Professor Gary Lu; (**l**) Professor Shen Xueyong (left). Beijing, China, 2–4 December 2017.

**Figure 32:**
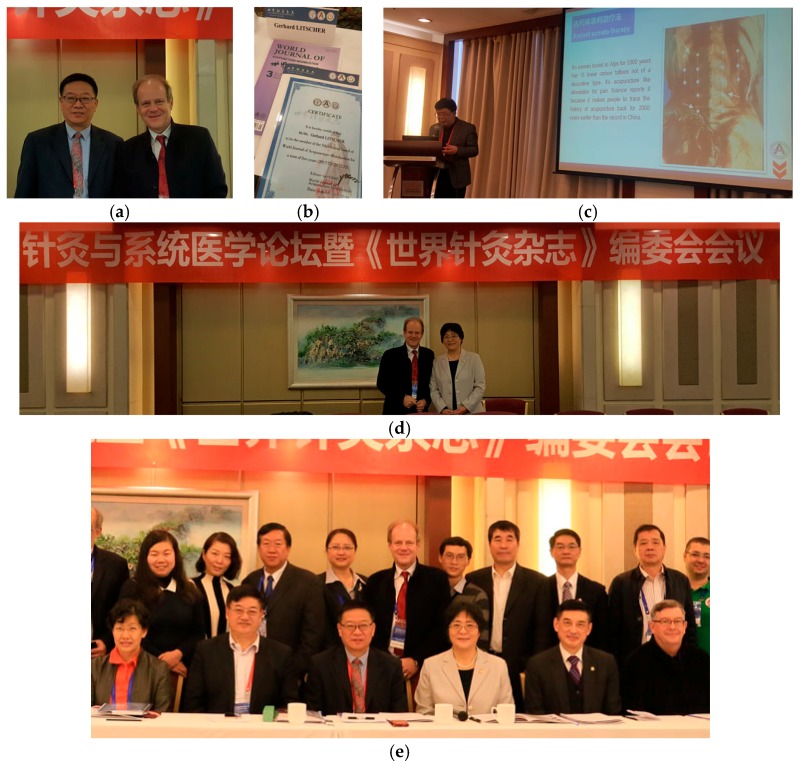
(**a**–**e**) Fifth (2017—2021) Editorial Board of the World Journal of Acupuncture (WJAM): Moxibustion. (**a**) Professor Yu Xiaochun, Editor-in-chief of WJAM and Professor Gerhard Litscher (right); (**c**) Professor Zhu Bing, former Director of the Institute of Acupuncture and Moxibustion at China Academy of Chinese Medical Sciences; (**d**) Professor Liu Weihong, Deputy Editor-in-chief and Professor Gerhard Litscher (left), member of the editorial board; (**e**) Part of the editorial board. Beijing, China, 4 December 2017.

**Figure 33:**
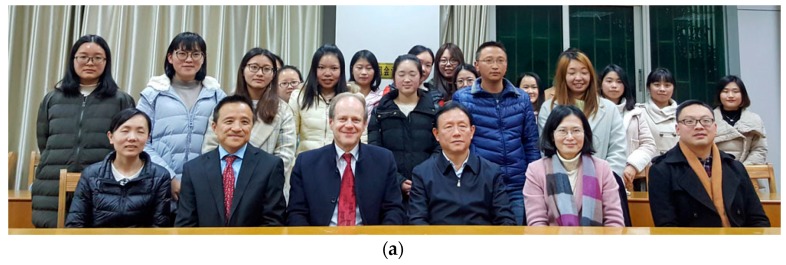

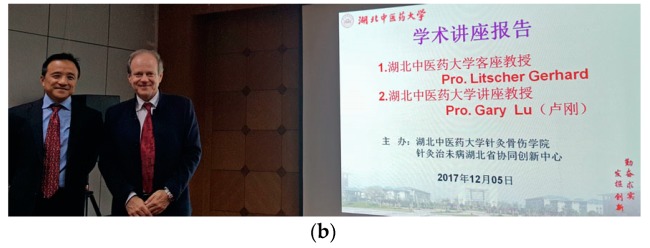
Hubei University of Chinese Medicine. (**a**) First row from left to right: Professor Gary Lu (2nd from left), Professor Gerhard Litscher, Professor Wang Hua, and Professor Liang Fengxia; (**b**) Professor G. Lu (left) and Professor G. Litscher. Wuhan, China, 5 December 2017.

**Figure 34:**
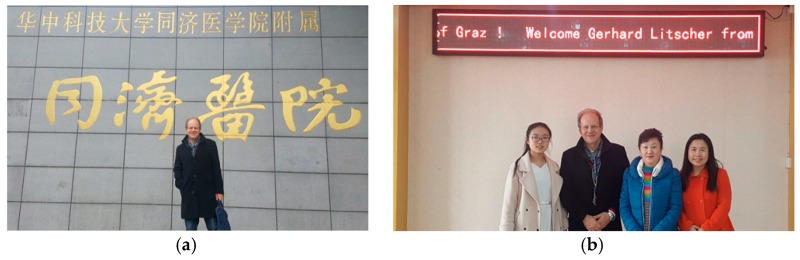
(**a**,**b**) Huazhong University of Science and Technology, Tongji Medical College. Professor G. Litscher with representatives from the School of Nursing. Wuhan, China, 6 December 2017.
